# Turbo FISH: A Method for Rapid Single Molecule RNA FISH

**DOI:** 10.1371/journal.pone.0075120

**Published:** 2013-09-16

**Authors:** Sydney M. Shaffer, Min-Tzu Wu, Marshall J. Levesque, Arjun Raj

**Affiliations:** Department of Bioengineering, University of Pennsylvania, Philadelphia, Pennsylvania, United States of America; Instituto de Medicina Molecular, Portugal

## Abstract

Advances in RNA fluorescence *in situ* hybridization (RNA FISH) have allowed practitioners to detect individual RNA molecules in single cells via fluorescence microscopy, enabling highly accurate and sensitive quantification of gene expression. However, current methods typically employ hybridization times on the order of 2–16 hours, limiting its potential in applications like rapid diagnostics. We present here a set of conditions for RNA FISH (dubbed Turbo RNA FISH) that allow us to make accurate measurements with no more than 5 minutes of hybridization time and 3 minutes of washing, and show that hybridization times can go as low as 30 seconds while still producing quantifiable images. We further show that rapid hybridization is compatible with our recently developed iceFISH and SNP FISH variants of RNA FISH that enable chromosome and single base discrimination, respectively. Our method is simple and cost effective, and has the potential to dramatically increase the throughput and realm of applicability of RNA FISH.

## Introduction

Over the past several years, the emergence of new single cell gene expression measurement techniques have revealed that levels of gene expression can vary hugely from cell to cell [1], [2]. These methods include those that are protein-based, such as GFP and immunofluorescence, and those that are nucleic acid based, including single-cell RT-qPCR [3]–[6], digital RT-PCR [7], single-cell sequencing [8] and single molecule RNA fluorescence *in situ* hybridization (single molecule RNA FISH).

Single molecule RNA FISH offers a number of advantages over other single cell expression quantification tools. In its latest incarnation, it offers the ability to detect individual RNA molecules via fluorescence microscopy, in which each RNA molecule appears in the cell as a bright, diffraction limited spot [9], [10]. Using software to count the spots, one can quantify the absolute number of RNA in individual cells without requiring any amplification, even within the cell’s natural developmental context [10], [11]. Moreover, one can analyze spot positions to gain insights into the location of RNA within the cell [12], [13]. Examples include transcriptional dynamics at the site of gene [14], [15], motion at the site of transcription itself [16], [17], and viral RNA localization within the cell [18], [19].

RNA FISH does, however, suffer from some important drawbacks compared to other methods in its current incarnation. One is that it is typically a low-throughput method in the sense that, like RT-qPCR, one can usually only analyze around 5 or so genes at a time, although barcoding schemes can increase this number to many dozens and potentially hundreds [20]. Yet another issue is that most current protocols rely on a long hybridization (often overnight) and series of washes in order to generate adequate and specific signals. The latter limitation hinders the use of RNA FISH in many scenarios, as it is considerably slower than RT-qPCR in practice, which usually takes on the order of hours to complete. The lack of a rapid version of RNA FISH also places severe restrictions on its use in diagnostic applications, in which timely results are hugely important.

We here describe a protocol that enables one to obtain quantifiable single molecule RNA FISH signals in under 5 minutes. We optimized both fixation conditions and hybridization conditions to achieve these results, showing there is a tradeoff between hybridization speed and probe concentration. We showed that these conditions apply across a variety of probes and cell types, and show that the technique is also compatible with our recently developed SNP FISH [21] and iceFISH [14] methods.

## Results

### RNA FISH Enables Single Molecule Detection

**Figure 1:**
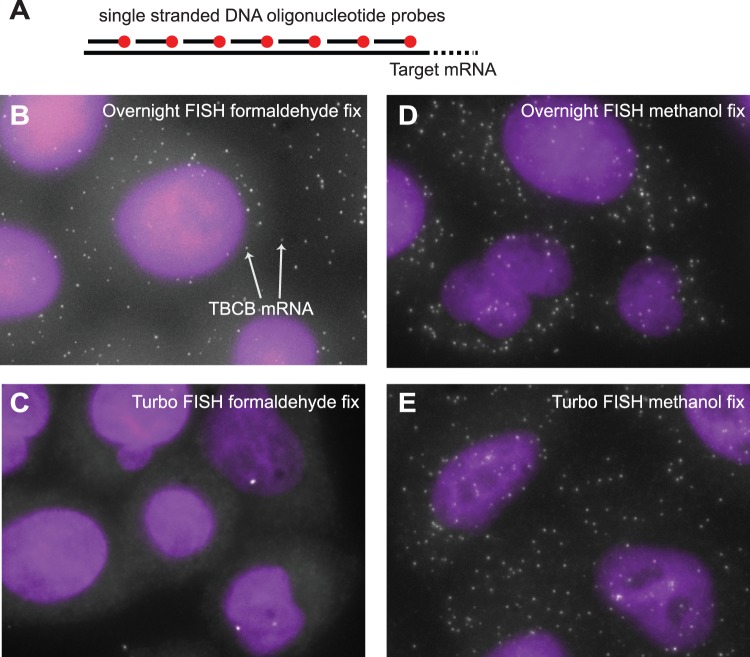
Depiction of the RNA FISH scheme and demonstration of rapid hybridization. A. Schematic of the single molecule RNA FISH method, in which we use dozens of short fluorescently labeled oligonucleotides that all target the same RNA molecule. B. Image showing RNA FISH targeting mRNA from the *TBCB* gene under standard overnight hybridization conditions (formaldehyde fixation). Each spot is a single mRNA molecule. C. Image showing RNA FISH signals from an attempt at rapid hybridization (5 minutes) with a high concentration of probe but with formaldehyde fixation. D., E. Traditional overnight hybridization and Turbo RNA FISH hybridization using methanol-fixed cells. All images are maximum projections of a stack of optical sections encompassing the three-dimensional volume of the cell. DAPI (nuclear stain) is in purple.

The method we employ for RNA FISH involves the use of several 20-base long single-stranded DNA oligonucleotides, each individually labeled [10], [22] (Fig. 1A). We design these oligonucleotides to bind to different segments of the target RNA via Watson-Crick base pairing, and the combined fluorescence from all the fluorophores at the single RNA leads to a fluorescent spot of intensity much higher than that of the background; we show a representative image for a probe targeting *TBCB* mRNA in Fig. 1B).

### Fixation Conditions

Traditionally, we have performed our hybridizations overnight in order to obtain strong signals. In order to perform rapid RNA FISH, we initially reasoned that one could speed the hybridization kinetics by increasing the concentration of probe included in the hybridization. Thus, we initially attempted to speed hybridization by simply increasing the amount of probe in our hybridization solution. We found, however, that despite increasing the concentration 20 fold, the signals were greatly diminished at hybridization times of 5 minutes (Fig. 1B,C). Our normal protocol utilizes cells that are fixed with formaldehyde, and we wondered whether the cross-links created by this form of fixation could impede the ability of the oligonucleotide probes to find their targets. To investigate this possibility, we performed hybridization with both ethanol- and methanol- fixed cells (each performed at −20C), both of which do not generate cross-links. We found that both alcohol-based fixatives performed considerably better (Fig. 1D, E), generating images that were roughly equivalent to those obtained by overnight hybridization with standard conditions. (We note also that we reduced the washing time in these cases to three one-minute washes, for a total of 8 minutes.).

**Figure 2:**
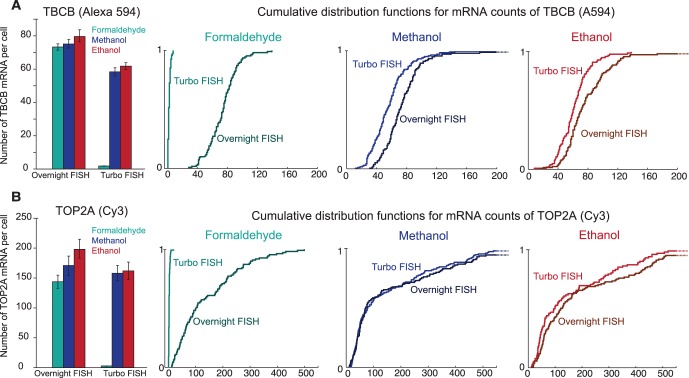
Comparison of fixation conditions for both traditional overnight hybridizations and rapid hybridization. A. Comparison of number of spots detected and cumulative distribution functions for the *TBCB* gene with probes labeled with the Alexa 594 fluorophore. Error bars represent the standard error of the mean. No statistically significant differences exist between the overnight RNA FISH samples. Turbo RNA FISH for *TBCB* gene on formaldehyde-fixed cells is statistically different from Turbo RNA FISH on methanol- and ethanol-fixed cells (p = 3.82×10^−65^ and p = 4.89×10^−96^, respectively; two-tailed t-test). For all conditions, we analyzed spot counts on 100–150 cells. B. Comparison of number of spots detected and cumulative distribution functions for the *TOP2A* gene with probes labeled with the Cy3 fluorophore. Error bars represent the standard error of the mean. Overnight RNA FISH for *TOP2A* gene on formaldehyde-fixed cells is statistically different from overnight RNA FISH on ethanol-fixed cells (p = 0.0067; two tailed t-test). No other statistically significant differences exist between overnight RNA FISH samples. Turbo RNA FISH for *TOP2A* gene on formaldehyde-fixed cells is statistically different from Turbo RNA FISH on methanol- and ethanol-fixed cells (p = 9.57×10^−28^ and p = 4.22×10^−30^, respectively; two-tailed t-test). For all conditions, we analyzed spot counts on 100–150 cells. Data shown represents one of two replicate experiments.

We then quantified the number of mRNA detected in all conditions using software similar in principle to that we have applied previously [10]. We found that after performing overnight hybridization, we obtained roughly the same number of RNA per cell with all fixation methods, but for rapid hybridizations performed at high concentrations, both alcohol-based fixatives gave similar results to those obtained from the overnight hybridizations, whereas the formaldehyde fixed cells performed much more poorly (Fig. 2A, B). We note, however, that the ethanol-fixed cells tended to disintegrate after spending over 48 hours in ethanol solution, so we used methanol-fixed cells for the rapid hybridization experiments in the remainder of the paper.

### Relationship between Concentration and Hybridization Time

**Figure 3:**
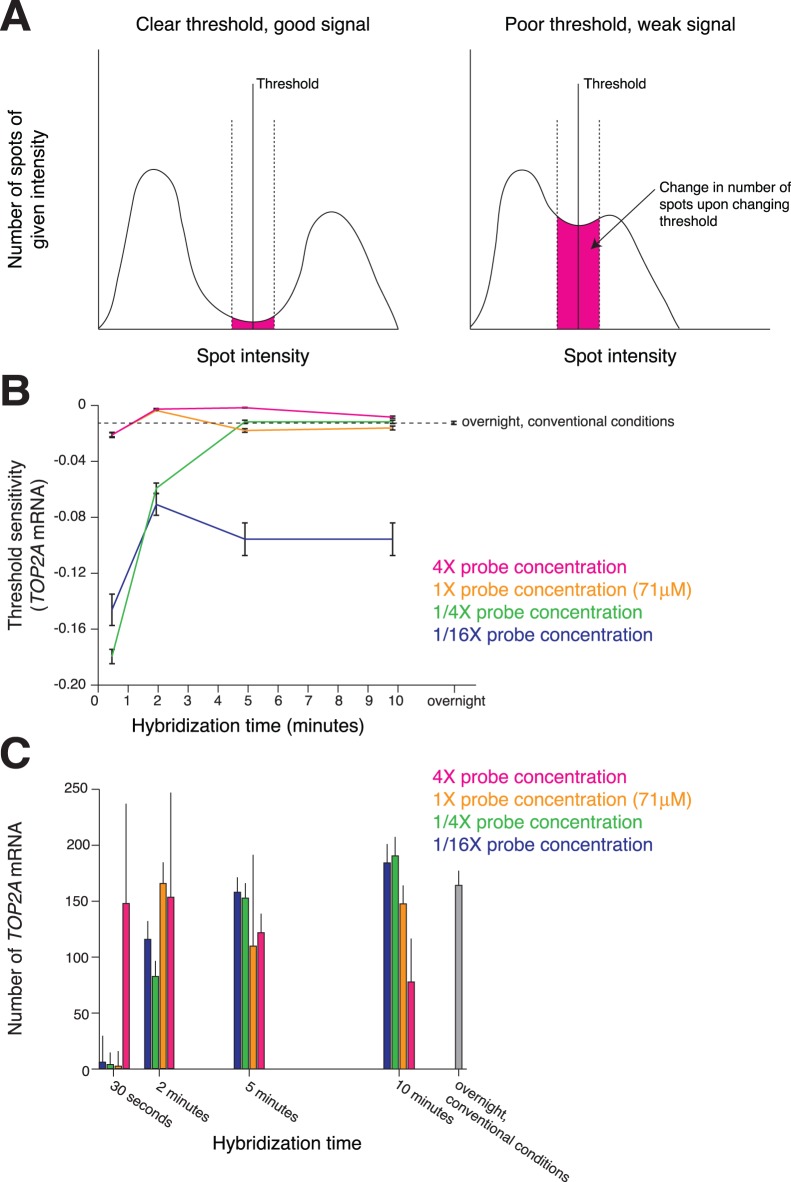
Quantification of signal quality and comparison of different hybridization times and probe concentrations. A. Schematic depicting the manner in which we quantify signal quality via threshold sensitivity. B. Sensitivity of threshold measured in varying probe concentrations and hybridization times. The dotted line represents the sensitivity of a traditional overnight RNA FISH. Error bars reflect standard error of the mean. C. Spot counts for the same conditions as in B. Error bars reflect standard deviation. At 10 minutes and for all probe concentrations, the spot counts for Turbo FISH are statistically different from overnight FISH (4X: p = 9.87×10^−6^, 1X: p = 0.0136, 1/4X: p = 4.86×10^−6^, 1/16X: p = 1.75×10^−11^; two-tailed t-test). For all conditions, we analyzed spot counts and calculated the sensitivity on 80–120 cells. Data shown represents one of two replicate experiments.

We then explored the degree to which there is a tradeoff between increasing the concentration of the probe and the hybridization time for rapid hybridization in methanol-fixed cells. In order to do so, we needed a means to assess and compare the quality of the signal in these various conditions. Ultimately, we settled on a metric based on the sensitivity of the threshold between signal and background (Fig. 3A). Briefly, we first use a linear filter designed to enhance spot-like signals. We then found all candidate spots by locating all regional maxima. These candidate spots consist of two populations, one corresponding to background spots and one corresponding to the target RNA molecules. When the signals are clear and quantifiable, the intensities of the RNA spots should be nicely separated from those of the background spots (Fig. 3A). However, if the RNA spots are not of high quality, then the spot intensities of the two populations can blend together, making it difficult to accurately quantify the number of true RNA spots within the image (Fig. 3A). To quantify this difference, we measured the degree of separation in the intensities of the two subpopulations by essentially measuring the sensitivity of the threshold separating the two; i.e., once the threshold is set, if we move the threshold slightly higher or lower, we measured the relative change in the number of RNA detected (Fig. 3A). We found that this metric for quantification captured the qualitative visual differences between conditions. We further note that metrics such as spot intensity and average spot count can be somewhat misleading as metrics of the ability to accurately count RNA in single cells (Fig. 3C). For instance, we have found that RNA spot intensity in and of itself need not be particularly high for accurate spot counting; rather, it just needs to be clearly and uniformly higher than the intensity of background spots. Average spot counts are also problematic because even when thresholds are ill-defined (as in Fig. 3A, right), one could still choose thresholds that yield similar spot counts on average, even though another person might equally well choose a different threshold, giving completely different results. For these reasons, we primarily focused on the sensitivity metric as an objective metric of signal quality.

We here present data from A549 cells, a common cancer cell type that we have found overall to be more difficult to perform rapid hybridizations in (hence providing a stringent test of our method). We performed RNA FISH (targeting TOP2A mRNA) over a range of hybridization times from 30 seconds to 10 minutes and probe concentrations ranging from our conventional probe concentration to 100 fold greater (approximately 4.4 µM to 400 µM). Throughout, we compared also to our traditional overnight hybridization protocol (Fig. 3B, C). We found that we were able to obtain readily quantifiable signals after 5 minutes of hybridization in the A549 cells (Fig. 3B, C). We found that there is a clear tradeoff in that higher concentrations of probe in the hybridization solution allow for shorter hybridization times. The exact amount of time and concentration one should use in these cases will of course depend on the constraints of the problem at hand, but we believe that a 5-minute hybridization at a probe concentration of 500 µM would be practical in many real-world scenarios. We also note that it is in some cases possible to perform rapid hybridizations in as little as 30 seconds with high concentrations of probe.

**Figure 4:**
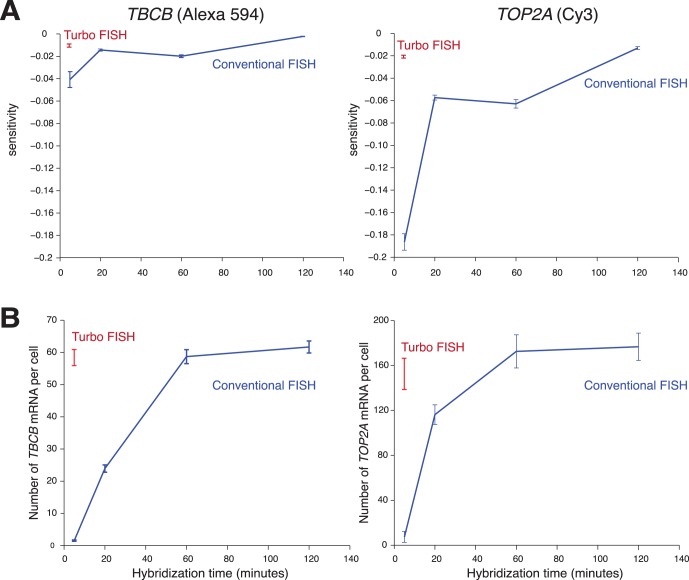
Comparison of signal from Turbo RNA FISH (5 minutes; red) to conventional RNA FISH (blue). A. Comparison of RNA FISH signal sensitivity at a range of hybridization times. Error bars reflect standard error of the mean. At 5 minutes, we found a statistically significant difference in signal sensitivity between Turbo FISH and conventional FISH for *TBCB* gene and *TOP2A* gene (p = 4.75×10^−11^ and p = 1.19×10^−74^, respectively; two-tailed t-test). B. Comparison of RNA FISH spot count at a variety of hybridization times. Error bars reflect standard deviation. At 5 minutes, we found a statistically significant difference in RNA FISH spot count between the Turbo FISH and conventional FISH for *TBCB* gene and *TOP2A* gene (p = 1.69×10^−68^ and p = 2.07×10^−20^, respectively; two-tailed t-test). For all conditions, we analyzed spot counts and calculated sensitivity on 100–150 cells. Data shown represents one of two replicate experiments.

For comparison, we also performed the same analysis by using the concentrations and wash protocol we used for our conventional overnight RNA FISH, except performing the hybridization for various amounts of time. We found that we obtained poorly quantifiable signals (as indicated by the sensitivity metric) once the hybridization time went below 2 hours, which is 24-fold as much time as our rapid hybridization assay (Fig. 4A, B).

### iceFISH and SNP FISH

**Figure 5:**
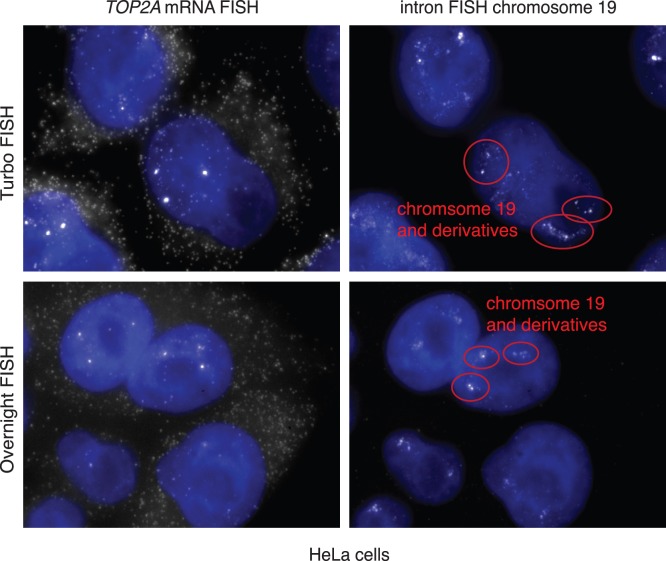
Demonstration of Turbo iceFISH. We performed Turbo FISH using iceFISH probes that targeted a total of 20 introns in genes on chromosome 19 (right panels), while simultaneously performing RNA FISH for TOP2A mRNA (left panels). We compared both Turbo FISH to conventional RNA FISH performed overnight (top vs. bottom panels). All images are maximum projections of a stack of optical sections encompassing the three-dimensional volume of the cell. DAPI (nuclear stain) is in blue.

In our lab, we have recently developed two variants of single molecule RNA FISH: 1. a method based on targeting introns that reveals chromosome structure and transcriptional activity (intron chromosomal expression FISH or iceFISH [14]), and 2. a method that utilizes both a new probe design and spot colocalization analysis to enable us to detect single nucleotide differences on individual transcripts (SNP FISH [21]). We wanted to test whether these methods would work in the rapid hybridization format. For iceFISH, we constructed an intron-based chromosomal “paint” that targets chromosome 19. We found that the iceFISH signals were comparable to those obtained via conventional overnight FISH using our rapid hybridization conditions (Fig. 5).

**Figure 6:**
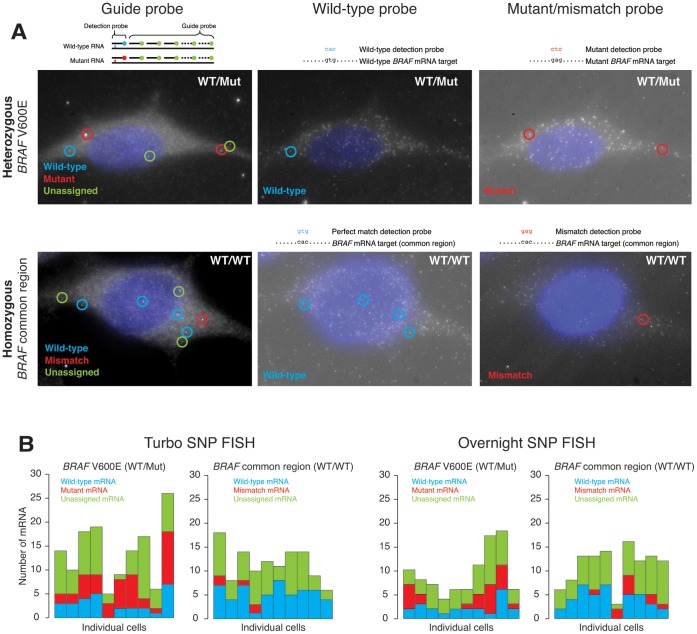
Demonstration of Turbo SNP FISH. A. Demonstration of SNP FISH efficacy under Turbo FISH and conventional RNA FISH conditions in WM983b cells. We targeted BRAF mRNA with guide probes, and then used detection probes that targeted either the V600E mutation for which BRAF is heterozygous in this cell line (top panels) or a common region for which BRAF is homozygous in this cell line (bottom panels). Left panels show the signals from the guide probe (that labels the mRNA), the middle panel shows the detection probe that detects the wild-type sequence, and the right panel shows the detection probe that detects the mutant sequence. B. Quantification of RNA as being either mutant or wild type in this cell line. Each bar corresponds to data from a single cell.

For SNP FISH, we used an approach in which we use a single oligonucleotide “SNP detection” probe hybridized to a “mask” oligonucleotide that leaves just a short “toehold” region available to nucleate binding to the target RNA (Fig. 6). The toehold region is short enough (5–10 bases) that it provides discrimination of single-base mismatches, but upon the binding of the correct probe, the mask dissociates via strand displacement [23], leading to the formation of a long (∼20–30 base) hybrid that provides stability. Meanwhile, we labeled the rest of the target RNA using conventional RNA FISH probes (which we call “guide probes”) that tell us where the target RNA are within the cell. Using colocalization between the SNP detection probe and the guide probe, we could assign each RNA based on whether or not it has the SNP. Our previous work demonstrated that this approach works under conventional RNA FISH conditions [21]. To check whether we were able to perform SNP FISH in rapid hybridization conditions, we used higher concentration of probes and shortened hybridization times (5 minutes) in methanol fixed cells. We tested Turbo SNP FISH in WM983b cells (gift of Meenhard Herlyn, Wistar Institute), which are heterozygous for the V600E mutation in the BRAF gene. We used both probes targeting the V600E BRAF mutation or a region common to both alleles on the BRAF mRNA as a control for non-specific binding (Fig. 6A). We found that in both Turbo SNP FISH and conventional overnight SNP FISH, the probes targeting the heterozygous base in BRAF indeed showed roughly equivalent levels of both mutant and wild-type mRNA (Fig. 6A, top). The probes targeting the region common between the two alleles identified virtually all the mRNA as being wild-type in both turbo and conventional conditions, showing that the rate of cross-hybridization remained low even with rapid hybridization conditions (Fig. 6A, bottom). Quantitatively, the results from both turbo and conventional SNP FISH were similar, both to each other and to our previous results [21] (Fig. 6B).

## Discussion

In this paper, we have described a protocol that enables rapid and quantitative detection of RNA targets via RNA FISH. We found that alcohol-based fixatives provide the necessary probe accessibility for rapid hybridization via increased probe concentration, potentially enabling hybridizations in as little as 30 seconds.

Our experiments show that there is a straightforward tradeoff between concentration of probe and the speed of hybridization. We have found that increasing probe concentration by 20X compared to our normal overnight protocol yields reliable RNA FISH results after just 5 minutes of hybridization. At first glance, this increased probe concentration may not seem economically viable, considering the increased use of probes (which are the most costly reagent in the RNA FISH protocol). However, we note that because of the decreased time for drying, our protocol uses roughly 10 fold less hybridization solution for the hybridization itself, greatly mitigating such concerns. We believe that the ultimate choice of how much probe to use and how fast to drive the reaction will depend on the specifics of the application at hand. In some cases, getting a hybridization time of 5–10 minutes may be perfectly fine, in which case one may not need to use large concentrations of probe. However, in some situations, such as during a surgical procedure, the decreased hybridization times may be a benefit that outweighs the cost of increased probe usage.

Of course, even with rapid hybridizations, we have not addressed the issue of the imaging time itself. Typically, image acquisition may require taking image stacks from multiple positions on the slide to obtain enough cells’ worth of image data to make statistically significant claims about differences in gene expression. Currently, doing so could take on the order of 10–20 minutes per condition. However, we believe that technical advances can reduce the time required for both image acquisition and analysis by at least an order of magnitude. In such a case, one could envision comparing gene expression in two samples in well under 30 minutes from living cells to quantified data.

It may be useful here to make a comparison to other methods such as RT-qPCR. RT-qPCR is the current gold standard for gene expression analysis, widely considered to be the most accurate method for quantifying gene expression to date. It has many benefits, including high dynamic range, low cost per reaction, and the ability to parallelize in 96-well plate format. The qPCR itself usually takes on the order of 1–2 hours to complete, but if one includes both RNA extraction and setup time, the total time required is probably closer to around 3–4 hours. (These extra steps also increase the cost of the experiment as well.) We believe that with rapid hybridization, RNA FISH competes favorably with RT-qPCR on most counts. With respect to quantification, our method provides accurate, absolute counts of gene expression of 3 to 5 genes in individual cells without the explicit need for normalization. Since RNA FISH is a direct detection scheme without any amplification, we are able to detect even small fold-changes with high precision, differences that would be hard to measure accurately with RT-qPCR, at least not without a large number of replicates. The cost per reaction is probably dominated by the cost of the probe, which is currently around $300–$600 per probe set for 10,000 hybridizations ($0.06 per reaction) and is thus comparable to a molecular beacon or Taq-man RT-qPCR probe. Of course, costs of labor, equipment and other reagents are variables that are hard to predict, but will be of the same order of magnitude, although we note that the labor required for RNA FISH is probably lower, whereas the cost of an automated microscope is admittedly higher than most qPCR machines. Both the accuracy and cost comparisons to RT-qPCR were valid even with overnight RNA FISH.

The time required for previous iterations of RNA FISH, however, was considerably longer than for RT-qPCR, and our new method alleviates that discrepancy. If one is just comparing the expression of a few genes in a few conditions, then we believe our method is unequivocally several times faster than RT-qPCR, especially when one factors in RNA extraction and setup time. For analyzing larger numbers of genes in parallel, though, the imaging time will become a factor. If one assumes 5–10 minutes per condition and triplex RNA detection, then analyzing, say, 20–30 genes could require up to 2 hours. With advances in high throughput imaging, we anticipate that one could reduce this time by an order of magnitude, thus further increasing the speed advantages.

Another major advantage of RNA FISH is that it also provides single cell information, something that is much more difficult to obtain with single cell RT-qPCR approaches. This enables one to measure variability in gene expression from cell to cell. Since the measurements yield absolute numbers of RNA, the measurements do not necessarily require normalization to an internal control (such as GAPDH), although one could perform such an analysis if one wished through multiplexing. Normalization can be difficult to perform with RT-qPCR approaches, since one typically uses all the material for a single qPCR reaction, leaving none for further normalization.

Furthermore, RNA FISH also provides spatial information on the localization of RNA. Such information is important both for examining differences from cell to cell within a tissue and even subcellular spatial localization. In tissues, one can easily identify particular cells by labeled RNA specific to those cells with one color and then looking at the gene of interest in another color. Subcellular information can be of particular importance for RNA that localize to particular regions of the cell, such as many non-coding RNA, in which case RNA FISH can reveal much about its behavior.

We have also shown that one can perform iceFISH and SNP FISH to visualize chromosomes and single base changes, respectively, with rapid hybridization. Such techniques could be useful for rapidly diagnosing chromosomal abnormalities and for rapid genotyping of particular single nucleotide variants.

In summary, our method for rapid hybridization results in orders of magnitude improvements in hybridization time for single molecule RNA FISH, enabling a new set of high throughput and rapid diagnostic applications.

## Methods

### Cell Culture

We cultured A549 cells (ATCC CCL-185), HeLa cells [24], and primary human foreskin fibroblasts (ATCC CRL-2097) in Dulbecco’s modified Eagle’s medium with Glutamax (DMEM, Invitrogen) supplemented with 10% fetal bovine serum and penicillin/streptomyocin. WM983b cells [25] were cultured in melanoma isolation media containing 80% MCDB153, 18% Leibovitz’s L-15, 2% fetal bovine serum, 1.68 mM CaCl_2_, and penicillin/streptomyocin.

### Formaldehyde Fixation

We fixed cells for 10 minutes in 4% formaldehyde/10% formalin in 1X phosphate buffered saline solution at room temperature. Following fixation, we washed cells twice with 1X phosphate buffer solution and then permeabilized the cells with 70% ethanol and stored them at 4°C for at least overnight.

### Alcohol Fixation

We fixed cells in pre-chilled ethanol or methanol (−20°C) for 10 minutes. Following fixation, we proceeded immediately to RNA FISH or Turbo RNA FISH.

### RNA FISH

To perform RNA FISH, we followed the protocol in Raj et al. Nat Meth 2008 [10] with minor modifications. We pre-washed cells with wash buffer containing 10% formamide and 2X saline-sodium citrate (SSC). We then performed hybridization by adding 1 µL of probe to 50 µL of hybridization buffer consisting of 10% formamide, 2X SSC, and 10% dextran sulfate (w/v). For the overnight hybridizations, the final probe concentrations was 3.5 µM for TOP2A probe and 4.9 µM for TBCB probe. We hybridized the samples overnight in a humidified chamber at 37°C. Following hybridization, we washed the samples twice with wash buffer for 30 minutes at 37°C. We then imaged the samples in 2X SSC.

### Turbo RNA FISH

For Turbo RNA FISH, we removed the alcohol from previously fixed samples and performed hybridization with 5 uL of hybridization buffer containing 71 µM TOP2A probe and 98 µM TBCB probe (unless otherwise specified), 10% formamide, 2X SSC, and 10% dextran sulfate (w/v). We hybridized the samples for 5 minutes (unless otherwise specified) on a covered hot plate at 37°C. Following hybridization, we washed the samples three times for one minute at 37°C with prewarmed wash buffer. We then imaged the samples in 2X SSC.

### Turbo iceFISH

For Turbo iceFISH, we followed the protocol of Levesque and Raj Nat Meth 2013 [14] but with methanol fixed cells, higher probe concentration and shorter hybridization times. We used the probes described in that publication to “paint” chromosome 19, with the chromosome paint labeled with Alexa 594. We performed iceFISH in HeLa cells, which have two normal copies of chromosome 19 and two derivative chromosomes, t(13;19) and t(6;19).

### Turbo SNP FISH

For Turbo SNP FISH, we followed the protocol of Levesque et al. Nat Meth 2013 [21] but with methanol fixed cells, higher probe concentration and shorter hybridization times. Notably, we also performed a 20-minute post-fix in formaldehyde after the hybridization to prevent probes from dissociating. We used the probes described in that publication to detect the BRAF V600E mutation, as well as one that targets a portion of BRAF that is the same on both alleles as a control. Our image analysis was the same as that described in the Levesque et al. Nat Meth 2013 [21] manuscript. We performed all the experiments in WM983b cells (gift of Meenhard Herlyn, Wistar Institute), which are heterozygous for the V600E mutation.

### Image Acquisition

We imaged all samples on a Nikon Ti-E inverted fluorescence microscope using a 100X Plan-Apo objective (numerical aperture of 1.43) and a cooled CCD camera (Andor iKon 934). We sequentially acquired three-dimensional stacks of fluorescence images in four different fluorescence channels using filter sets for DAPI, Cy3, Alexa 594, and Atto 647 N. Our exposure times ranged from 2–3 s for most of the dyes except for DAPI, for which we used ∼50 ms exposures. The spacing between consecutive planes in our stacks was 0.3 µm.The filter sets we used were 31000v2 (Chroma), 41028 (Chroma), SP102v1 (Chroma), a custom set from Omega as described previously [22], SP104v2 (Chroma) and SP105 (Chroma) for DAPI, Atto 488, Cy3, Alexa 594, Atto 647 N and Atto 700, respectively.

### Image Analysis and Quantification

After imaging, we then put our data through an image analysis pipeline for semi-automated spot recognition. We implemented the analysis pipeline in MATLAB. Briefly, our method for analysis involves running the images through a linear filter designed to enhance spots around the size of those we observe, then finding all regional maxima within the filtered image, and then counting the number of regional maxima below a variety of thresholds [10]. We then manually determine a threshold where the number of regional maxima changes the least upon changing the threshold (i.e., the number of spots is least sensitive to moving the threshold). To quantify sensitivity of the threshold, we took the derivative of the logarithm of the graph of the number of regional maxima below varying thresholds. We smoothed the derivative before quantifying to avoid noise due to local variations in the graph.
